# A new rare and endemic species of *Sloanea* (Elaeocarpaceae) from the Chocó region of Ecuador

**DOI:** 10.3897/phytokeys.160.54993

**Published:** 2020-09-08

**Authors:** Juan Ernesto Guevara Andino, Diana Fernandez-Fernandez

**Affiliations:** 1 Grupo de Investigación en Biodiversidad, Medio Ambiente y Salud-BIOMAS- Universidad de las Américas, Campus Queri, Quito, Ecuador Universidad de las Américas Quito Ecuador; 2 Keller Science Action Center, The Field Museum, 1400 South Lake Shore Dr., Chicago, IL 60605-2496, USA The Field Museum Chicago United States of America; 3 Instituto Nacional de Biodiversidad, Herbario Nacional del Ecuador QCNE, Quito, Ecuador Herbario Nacional del Ecuador Quito Ecuador

**Keywords:** Chocó, Ecuador, endemic, lowland humid forest, *
Sloanea
*

## Abstract

A new species collected in the lowland forests of the Chocó region of Ecuador, *Sloanea cayapensis*, is described and illustrated and its morphological similarities with other species of *Sloanea* are discussed.

## Introduction

The genus *Sloanea* L. is the second largest within the family Elaeocarpaceae and comprises approximately 200 species in the tropics and more than 120 species in the Neotropics (Smith 1954; Smith 2001; Sampaio 2009). Smith (1954) proposed that the centre of diversity for this group is the northern portion of the Amazon basin, including the Guiana Shield, but the genus is as diverse in Central and Western Amazonia as in the previously mentioned region (Castañeda 1981; Boeira 2010).

The most recent taxonomic study of *Sloanea* in Ecuador revealed that 25–30 species of the genus occur in the country (Jaramillo 2003, Pennington and Wise 2017) and this number has increased with recent new descriptions (Guevara Andino et al. 2016; Guevara Andino et al. 2017). The genus is very diverse in Amazon lowland forests and in the Chocó forests on Ecuador’s Pacific coast. As the southernmost extension of Colombia’s Chocó forests, Ecuador’s coastal Chocó is strikingly diverse (Gentry 1982) but remains poorly studied, despite several new species of the genus having been recently described (Palacios-Duque 2004a, b, 2005).

## Materials and methods

We describe a new species of *Sloanea* from the Ecuadorian Chocó region, based on an analysis of morphological characters from material deposited in four Ecuadorian herbaria: the Herbario de la Pontificia Universidad Católica del Ecuador (**QCA**), Herbario Nacional del Ecuador (**QCNE**), Herbario Alfredo Paredes (**QAP**) and Herbario Amazónico del Ecuador (**ECUAMZ**). We also compared the new species with images of type specimens deposited in JStor and reviewed voucher specimens in the virtual herbaria of the Field Museum (F), the New York Botanical Garden (**NY**), the Herbario Nacional de Colombia (**COL**) and the Herbário do Instituto Nacional de Pesquisas da Amazônia (**INPA**); herbaria abbreviations follow Thiers 2016. In this work, we used the sub-generic classification developed by Smith (1954) for the circumscription of the new taxa. Earlier works have demonstrated that a single diagnostic character, the position of the calyx in relation to the bud, is the only consistent character in the group and may be considered diagnostic for the circumscription of taxa to the subgenus level (Sampaio 2009; Guevara Andino et al. 2016).

## Taxonomic treatment

### Sloanea cayapensis

Taxon classificationPlantaeOxalidalesElaeocarpaceae

J.Jaram. & J.E. Guevara sp. nov.

6056567C-F2F2-5E19-9321-D037C398CDA7

urn:lsid:ipni.org:names:77211422-1

Figs 1 , 2 , 3 , 4

#### Diagnosis.

*Sloanea cayapensis* resembles *S. grandiflora* Smith and *S. fragrans* Rusby, the most morphologically similar taxa, but can be differentiated from them by having short petioles (2–7.5 cm), obovate-spatulate leaves, 6–8 free sepals, shorter thick and acute-obtuse anthers (1.5–2 mm), densely hirsute filaments and styles and by having capsules with large, flexible, curled bristles (2.8–6 cm).

**Figure 1.**
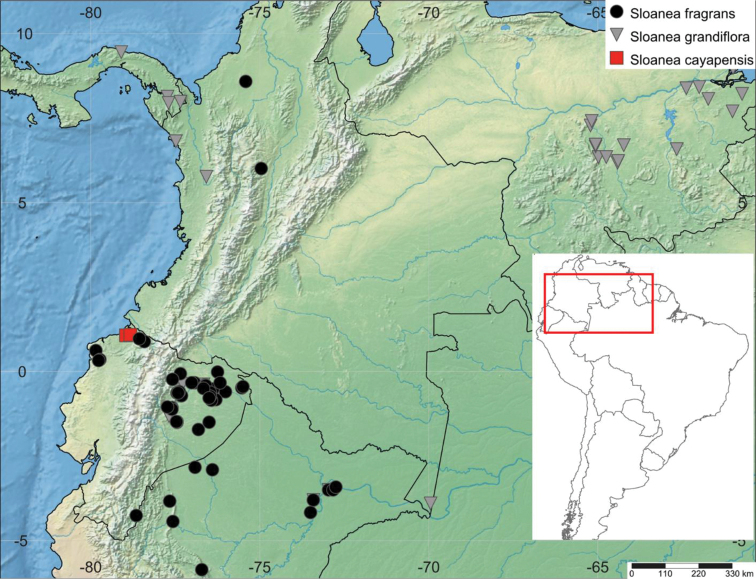
Map of the geographic distribution of *Sloanea cayapensis* J. Jaramillo ex J.E. Guevara, sp. nov. (red squares), *Sloanea fragrans* Rusby (black circles) and *Sloanea grandiflora* Sm. (grey inverted triangles).

#### Type.

Ecuador. Esmeraldas: Borbón-Río Cayapas, 78°50'W, 1°5'S, 10 m elev., 3 May 2003, *Jaime Jaramillo, A. Sola, S. Yandun 24200* (holotype [2 sheets]: QCA7007914! (fl, fr); QCA7007917!; isotypes QCA7007915!,QCA7007916! (fl), QCA204513!).

Medium-sized trees up to 15–18 m tall. Trunk striated, bark rough, brownish. Branchlets glabrous, quadrangular and covered by ovoid, cream-coloured lenticels. Leaves alternate; petioles 2–7.5 cm long, semi-terete, striated, shortly pubescent, thickened at the insertion with the blade; blades (18.8–)42–52(–61.8) cm long, (9.5–)17–26(–32) wide, coriaceous obovate-spatulate, attenuate-subcordate or rounded at base, obtuse-retuse at apex, the margins entire, slightly revolute; foliaceous stipules persistent at the top of the individual branchlets, 3.9–13 cm long, 2.1–3.9 cm wide, elliptical with acuminate apex, the margin entire or shallowly sinuate; primary vein prominent on the adaxial surface, very prominent and angular on the abaxial surface, secondary venation eucamptodromous, 11–20 secondary veins, prominent on the abaxial surface and ascendant (angle > 45°), flat on the adaxial surface, tertiary veins prominent on the abaxial surface, slightly flat on the adaxial surface. Inflorescence axillary, racemose; peduncles 2–6 cm long; rachis 5–18.5 cm long, slightly pubescent, deeply striated and quadrangular; pedicels 1–7 cm, stout, shortly pubescent, finely striate and quadrangular, pedicels, 1–1.5 cm long, navicular bracts at the base of individual pedicels, 4–4.5 mm long, dense appressed pubescence on both abaxial and adaxial surfaces, apex acute, commonly deciduous. Flowers with the receptacle large, expanded; sepals 6–8, free, 3–5 mm long, 2.5–3 mm wide, greenish coloured, ovate, apex acute-acuminate, margins entire, slightly involute, yellow on the outer surface, not covering the reproductive organs before anthesis. Stamens 5–6 mm long, yellow with orange anthers; filaments 3–3.5 mm long, densely hirsute, striated and angulate; anthers 1.5–2 mm long, densely hirsute, thick, the connective thin on the abaxial surface of the anther sacs, extended as an acute or obtuse awn, very short, up to 0.5 mm long; anther sacs not opening widely along entire length. Ovary 2–4 mm long, 1.5–2.7 wide, with four locules, 4-angled, ovoid, densely hirsute; placentation axillary; style to 8 mm long, densely hirsute at the base, becoming sparsely hirsute towards the apex. Fruits globose capsules 1.2–2.5 cm long, 1–2 cm wide, rounded, opening by 4 rigid valves; bristles 2.8–6 cm long, curled, contorted and flexible, laterally flattened, densely hirsute at the base, more sparse and appressed pubescence towards the apex, easily detached. Seeds not studied.

**Figure 2.**
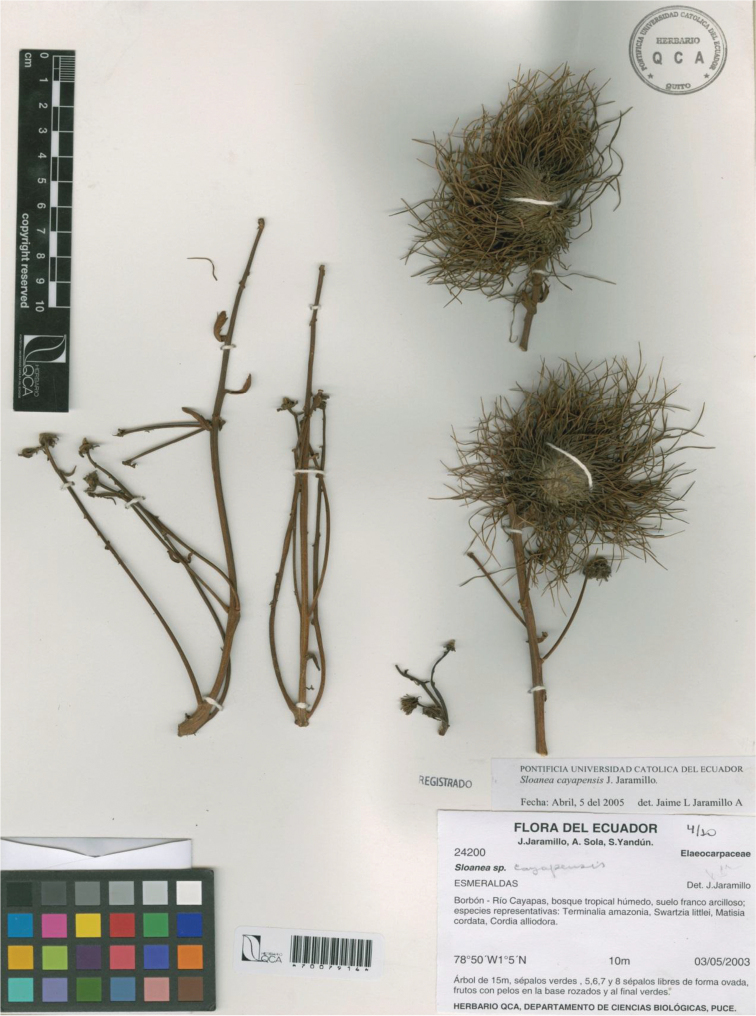
*Sloanea cayapensis*. Image from the holotype at QCA (*J. Jaramillo 24200*).

#### Specimens examined.

***Paratypes*.** Ecuador: Esmeraldas: La Chiquita, bosque secundario, 45 m elev., *Jaime Jaramillo* 24422 (QCA-236170!); Esmeraldas: Localidad Borbón, entre Punta Piedra y Maldonado, 78°58'W, 1°4'S, 60 m elev., febrero 13 de 1993, *Jaime Jaramillo 15016* (QCA-204514!).

#### Distribution and habitat.

*Sloanea cayapensis* is a medium-sized tree only known from two localities on high alluvial terraces along the Cayapas River in the Lowland Evergreen Forests of Equatorial Chocó (Ministerio del Ambiente del Ecuador 2013). This area lies in the Chocó floristic province, where forest structure is characterised by a canopy 25–30 m high, with occasional emergent trees reaching 40 m. High levels of endemism and dominance of families, such as Moraceae, Fabaceae, Meliaceae, Myristicaceae and Lecythidaceae, have been reported for this area (Gentry 1982; Ministerio del Ambiente del Ecuador 2013) and the label of the type specimen indicates that *S. cayapensis* co-occurs with the following tree species: *Terminalia amazonia* (Combretaceae), *Swartzia littlei* (Fabaceae), *Matisia cordata* (Malvaceae) and *Cordia alliodora* (Boraginaceae). The climate of this area is rainy near the Ecuador-Colombia border and becomes more seasonal to the south, where it transitions into the Lowland Seasonal Evergreen Forests of the Equatorial Chocó (Ministerio del Ambiente del Ecuador 2013).

**Figure 3.**
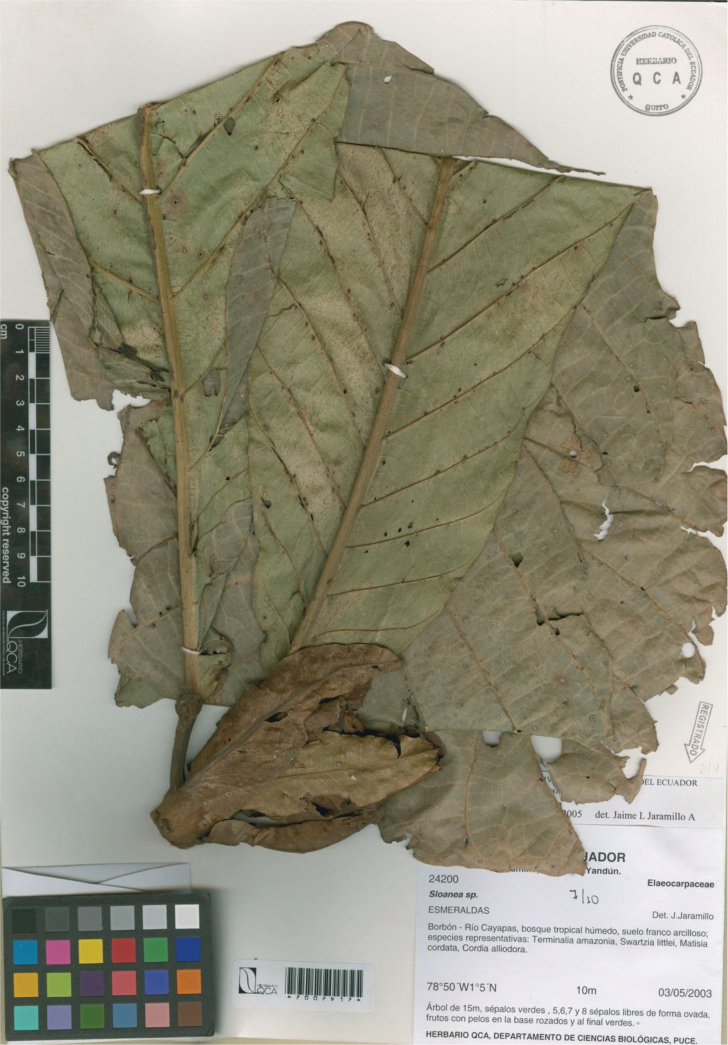
*Sloanea cayapensis*. Image from the holotype at QCA (*J. Jaramillo 24200*).

#### Etymology.

From the Spanish word Cayapa, with reference to the Chachi indigenous group that inhabits a great portion of the evergreen lowland forests of the Equatorial Chocó in Ecuador. The word Chachi means ‘pure’ in the Cha’palaachi language. The species name was first proposed by the late Ecuadorian botanist J. Jaramillo as a written annotation on herbarium specimens, but was never validly published.

#### Conservation status.

*Sloanea cayapensis* may be catalogued as Endangered (EN) following IUCN (2012) criterion A2c, which indicates: “An observed, estimated, inferred or suspected population size reduction of ≥ 50% over the last 10 years or three generations, whichever is the longer, where the reduction or its causes may not have ceased or may not be understood or may not be reversible” and criterion B1ab(iii), which indicates: “Extent of occurrence (EOO) estimated to be less than 5,000 km^2^ and estimates indicating continuing decline, observed, inferred or projected, in area, extent and/or quality of habitat”. The analysis using the GeoCat tool for geospatial conservation assessment determined an EOO of 2000 km^2^ (Bachman et al. 2011). *Sloanea cayapensis* is only known from two localities on the banks of the Cayapas River, in the lowland evergreen forest of the Equatorial Chocó. The area has suffered extensive clear-cutting in the last 30 years, leading to a drastic reduction of native forests and expansion of oil palm plantations and illegal logging, these being the major threats faced by the species. Since collection of the types in 2003, no other specimens of this species have been recorded despite the fact that subsequent field trips have sampled the same habitat occupied by this species. Over the last 26 years, the annual rate of deforestation in the lowland evergreen forest of the Equatorial Chocó in Ecuador has been 1.7–2.9% and the remnant forests in this area cover just 24% of their original extent (Sierra 2013). Consequently, the habitat occupied by this species in Ecuador has been drastically reduced. However, the species is likely to occur in similar habitats in the Colombian Chocó where the dynamic of deforestation is less drastic but a cause for concern.

**Figure 4.**
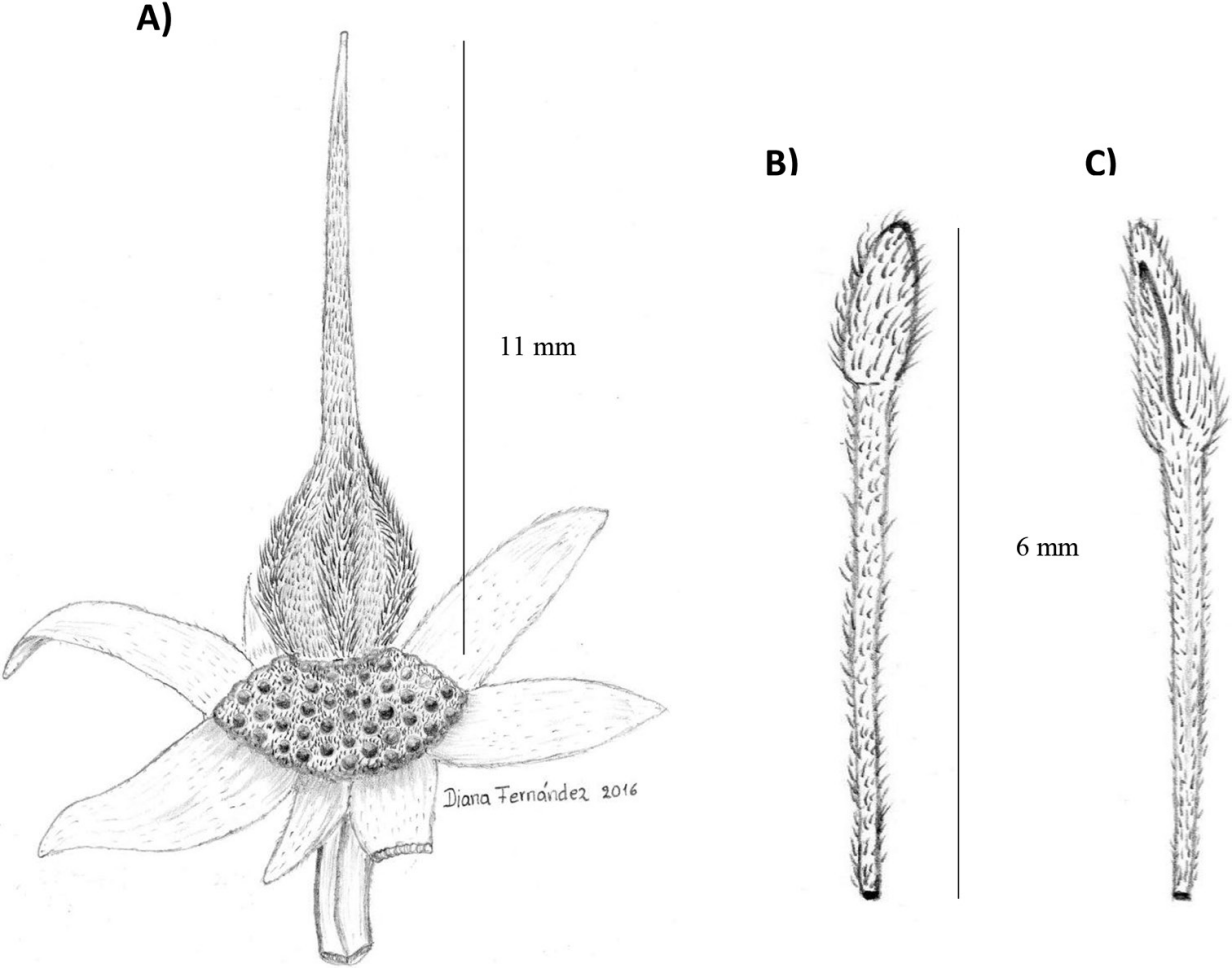
*Sloanea cayapensis***A** detail of ovary and sepals **B** dorsal view of stamen **C** ventral view of stamen (**A–C** from *J. Jaramillo 24200*QCA).

## Discussion

Based on our description, *Sloanea cayapensis* is morphologically similar to *S. grandiflora* and *S. fragrans*, both members of the subgenus Sloanea. The new species can be readily distinguished from *S. fragrans* by having longer (3–3.5 mm vs. 1.5–2 mm long) and densely hirsute angle-striated filaments (vs. cylindrical puberulent filaments). It also differs from *S. fragrans* by having shorter anthers (1.5–2 mm vs. 2–3 mm long), acute or obtuse awn-shaped (vs. lanceolate) anthers densely hirsute (vs. glabrous to sub-glabrous), lanceolate stipules with entire margins (vs. navicular with remarkable irregular serrate margins) and shorter petioles (2–7.5 cm vs. 5.5–20(–25) cm long) (see Table 1).

**Table 1.** Diagnostic characters for *Sloanea cayapensis* and its closest relatives, as well as their geographical distribution in the Neotropics: Western Amazon (WA), Central Amazon (CA), Chocó region (Chocó), Central America (CAm) and the Guiana Shield (GS).

Characters	*Sloanea cayapensis*	*Sloanea grandiflora*	*Sloanea fragrans*
Leaf size	(18.8–)42–52(–61.8) long, (9.5–)17–26(–32) cm wide	21–43.5 cm long, 15–25.5 cm wide	45–65 cm long, 21–29.5 cm wide
Leaf shape	Obovate-spatulate	Elliptic to elliptic-bovate	Obovate
Leaf margins	Entire to shallowly sinuate	Irregularly dentate	Irregularly undulate
Stipules	Lanceolate with entire margins	navicular with irregular serrate margins	navicular with finely dentate margins
Stamens	5–6 mm long	7–9 mm long	4–8 mm long
Filaments	3–3.5 mm long densely hirsute angle-striated	2–4 mm long angled or laterally flattened	1.5–2 mm long cylindrical puberulent
Anthers	1.5–2 mm long, thick and obtuse	3–4 mm long, linear-lanceolate	2–3 mm long, lanceolate
Ovary	Ovoid, 2–4 mm long, 1.5–2.7 mm wide	Ovoid, 4 mm long, 2.5 mm wide	Globose, 3 mm long, 3 mm wide
Style	Densely hirsute in its entire length up to 8 mm long	Pubescent at the base glabrous above, 7–9 mm long	Pubsecent in its entire length, 5–10 mm long
Capsule	Globose densely covered by curled flexible spines	Ellipsoidal covered by stramineous flexible curled spines	Globose densely covered by short spines
Elevation	0–500 m	0–1500 m	0–1100 m
Geographic distribution	Chocó	WA, CA, GS and CAm	WA and CAm

*Sloanea cayapensis* is distinguished from *S. grandiflora* in having a densely hirsute style (vs. glabrous style at the apex and slightly pubescent at the base), thin connective (vs. very wide connective between the anthers sacs on the abaxial surface), thick and obtuse anthers (vs. linear-lanceolate) and an ovoid and densely hirsute ovary with 4 locules (vs. pubescent ovary with 4–6 locules). It also differs from *S. grandiflora* in having lanceolate stipules (vs. navicular stipules with finely dentate margins), shorter pedicels (4–11 mm vs. 7–25 mm) and strongly ascendant numerous secondary veins (11–20 vs. 13–15 slightly ascendant secondary veins).

## Supplementary Material

XML Treatment for Sloanea cayapensis
